# Total thymectomy for thymic lymphoepithelioma-like carcinoma—report of two cases

**DOI:** 10.1186/s40792-019-0706-6

**Published:** 2019-10-26

**Authors:** Sachi Kawagishi, Naoko Ose, Masato Minami, Soichiro Funaki, Takashi Kanou, Kenji Kimura, Seiji Taniguchi, Eiichi Morii, Yasushi Shintani

**Affiliations:** 1 Department of General Thoracic Surgery, Osaka University Graduate School of Medicine, 2-2(L5), Yamadaoka, Suita-shi, Osaka 565-0871 Japan; 2 Department of Pathology, Osaka University Graduate School of Medicine, 2-2, Yamadaoka, Suita-shi, Osaka 565-0871 Japan; 3 Department of General Thoracic Surgery, Osaka University Graduate School of Medicine, 2-2, Yamadaoka, Suita-shi, Osaka 565-0971 Japan

**Keywords:** Thymic lymphoepithelioma-like carcinoma, Thymus, Total thymectomy, Masaoka staging system

## Abstract

**Background:**

Thymic carcinoma has been classified into 12 subtypes, thymic lymphoepithelioma-like carcinoma (LELC) is a type of them, and has a pathological organization similar to that of lymphoepithelioma, an undifferentiated type of nasopharyngeal carcinoma. According to a report from the International Thymic Malignancy Interest Group (ITMIG), thymic LELC is a rare tumor and accounts for 6% of all thymic carcinoma cases. We report two cases of surgical resection for thymic LELC and perform a search of other reports of thymic LELC, and clinical manifestations and follow-up data thus obtained are summarized.

**Case presentation:**

Two patients underwent surgical resection for thymic LELC. In both, tumors were detected in the anterior mediastinum and a total thymectomy was performed. Each was diagnosed with thymic LELC and classified in accordance with the Masaoka staging system as modified stage II. In recent examinations, one patient was doing well after undergoing total resection, whereas early recurrence of distant lymph node metastasis was noted in the other at 5 months after the total resection procedure and died thereafter from a different disease.

**Conclusion:**

We report two cases of surgical resection for thymic LELC. A successful total resection may positively affect prognosis: thus, long-term follow-up examinations must be performed.

## Background

Thymic lymphoepithelioma-like carcinoma (LELC), a thymic carcinoma that has been classified into 12 subtypes, has a pathological organization similar to that of lymphoepithelioma, an undifferentiated type of nasopharyngeal carcinoma [1]. Also like LELC of the head and neck, thymic LELC has been shown to have an association with Epstein-Barr virus (EBV) [2]. According to a report from the International Thymic Malignancy Interest Group (ITMIG), thymic LELC is a rare tumor and accounts for 6% of all thymic carcinoma cases [3], with affected patients generally showing a poor prognosis. Herein, we report details of 2 patients who underwent surgical resection for thymic LELC.

### Case reports

#### Case 1

**Fig. 1:**
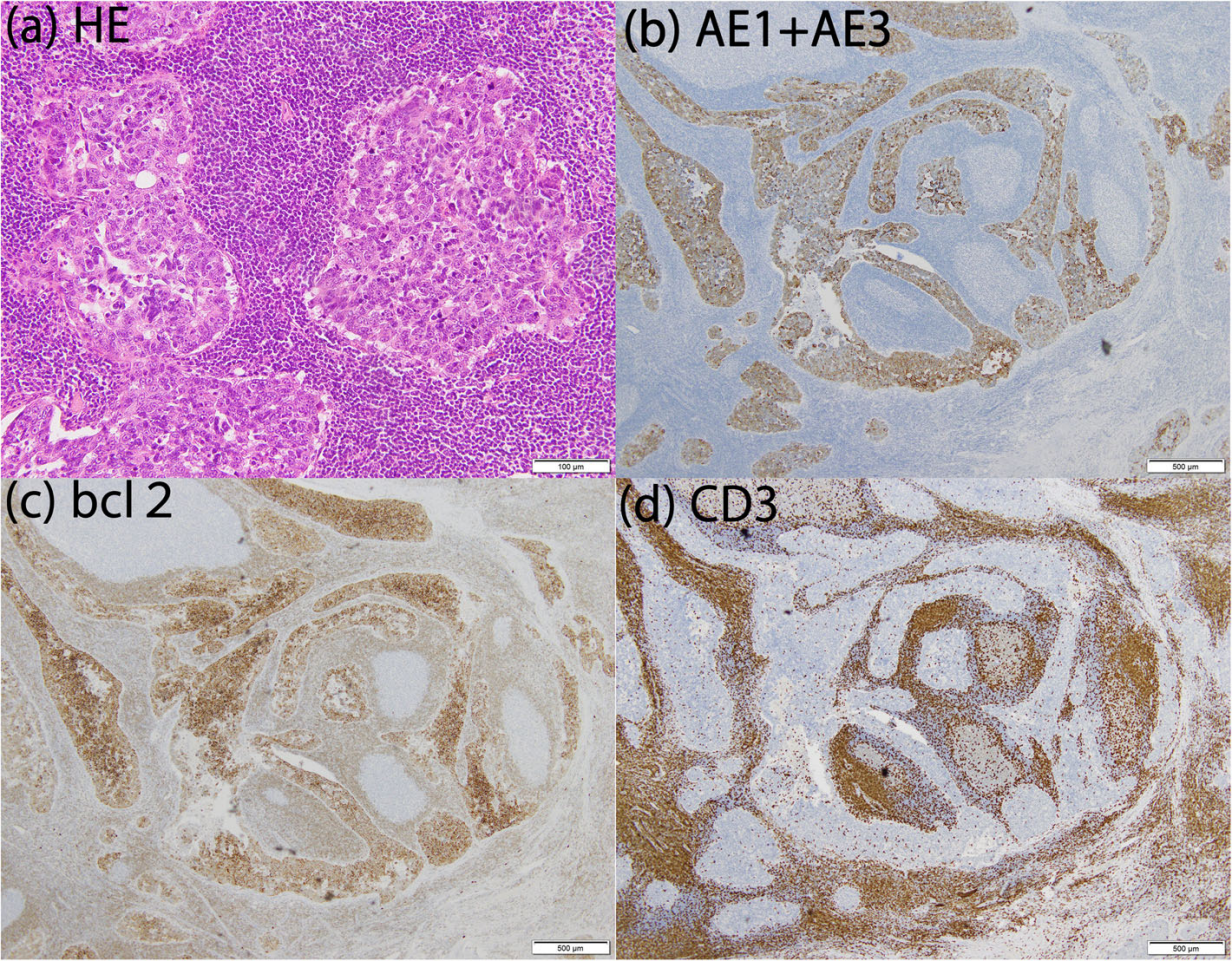
Case 1. Results of HE staining and immunohistochemical analysis. **a** HE staining showed that the tumor cells had edematous large and round nuclei and formed a solid corded net on the background of dense lymphocyte infiltration. Thymic disease was positive for **b** cytokeratin AE1 + AE3 and **c** Bcl2, while infiltrated lymphocytes were positive for **d** CD3 and CD5 and negative for CD99

A 65-year-old female presented with anterior chest pain and chest computerized tomography (CT) revealed a tumor in the anterior mediastinum. The size on CT images was measured at 36 mm, and there was no evidence of infiltration into adjacent tissue; thus, a thymoma was suspected. A surgical resection procedure was scheduled for diagnostic and therapeutic purposes. Partial resection of the thymus was performed using video-assisted thoracoscopic surgery and the intraoperative diagnosis was suspected thymoma. The tumor size was 33 × 28 × 20 mm, the operation time was 64 min, and the amount of bleeding was small. The postoperative pathological diagnosis was LELC. Histologically, tumor cells with edematous large and round nuclei formed a solid corded net on the background of dense lymphocyte infiltration. Immunohistochemically tumor cells were positive for Bcl2 and Cytokeratin AE1 + AE3, while infiltrated lymphocytes were positive for CD3 and CD5 and negative for CD99 (Fig. 1). Microscopic invasion into surrounding adipose fatty tissue beyond the capsular portion was noted. EB-encoded RNA in situ hybridization of the tumor was negative. We also performed 18F-fluorodexyglycose positron emission tomography/CT (FDG-PET/CT), with no abnormal accumulation seen to indicate distant metastasis.

Two months after the first operation, a total thymectomy and lymph node dissection were performed median sternotomy, with an operation time of 220 min and bleeding totaling 100 ml. Postoperative pathological findings revealed no tumor cells in the residual thymus and no lymph node metastasis. The TNM classification was p-T1aN0M0 stage I, while it was stage II in accordance with the Masaoka staging system. Ten months after the first operation, the patient had no recurrence and no adjuvant therapy was performed.

#### Case 2

**Fig. 2:**
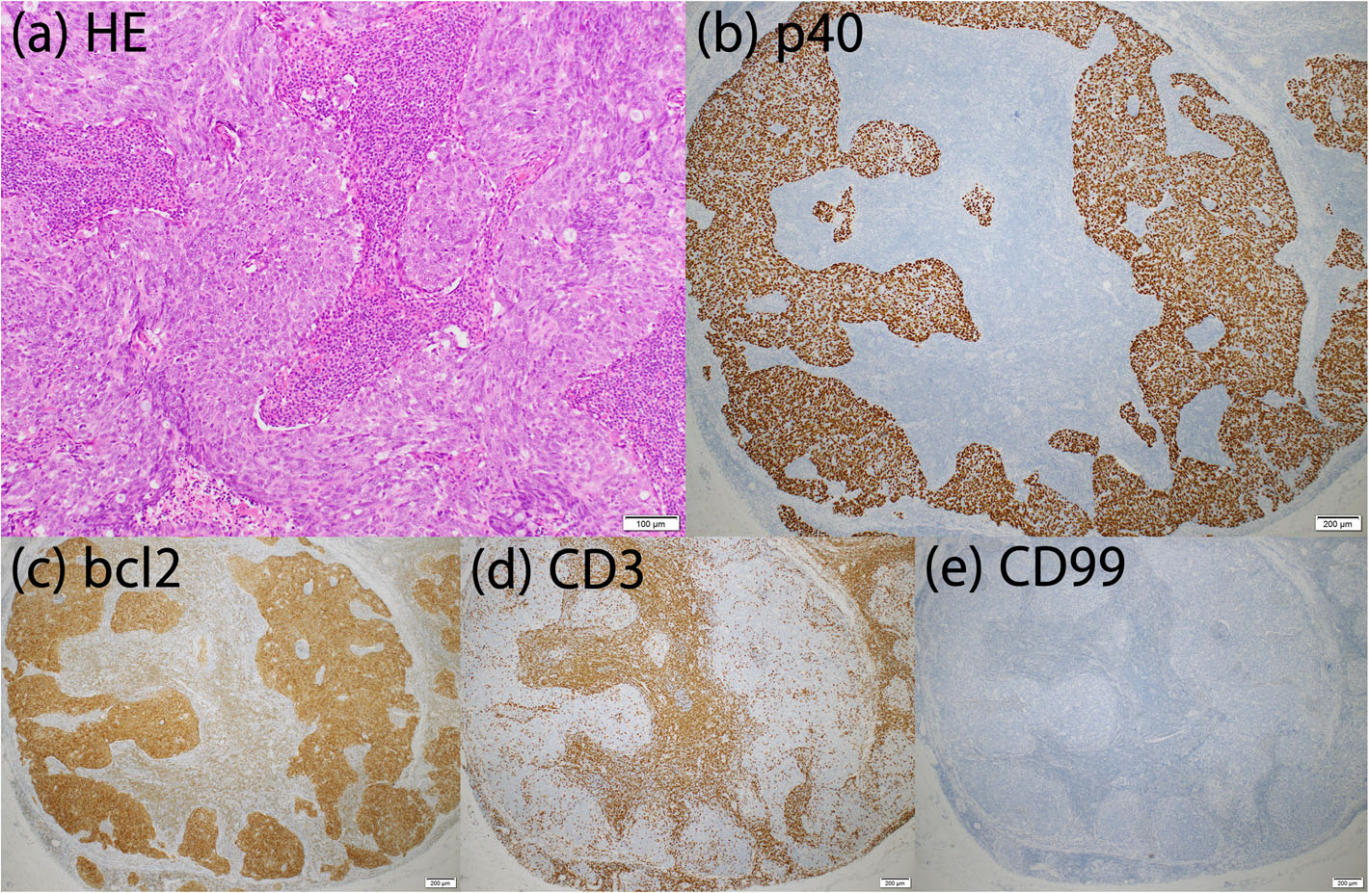
Case 2. Results of HE staining and immunohistochemical analysis. **a** HE staining showed that the tumor cells had edematous large and round nuclei and formed a solid corded net on the background of dense lymphocyte infiltration. Thymic disease was positive for **b** p40, and **c** Bcl2 and CD5, while infiltrated lymphocytes were positive for **d** CD3 and negative for **e** CD99

Chest X-ray findings in a medical checkup examination of an 81-year-old female showed an abnormal shadow. She came to our institution and chest CT results revealed a tumor in the anterior mediastinum. The size on CT images was 41 mm and there was no apparent infiltration of adjacent tissues; thus, a thymoma was suspected. An FDG-PET/CT examination revealed uptake (SUVmax 6.5) in the tumor. A total thymectomy and pretracheal lymph node dissection were performed by a median sternotomy combined with resection of the pericardium. The operation time was 114 min and bleeding was 50 ml, while the tumor size was 58 × 45 × 26 mm. Postoperative pathological findings revealed edematous large and round nuclei in the tumor cells, which formed a solid corded net on the background of dense lymphocyte infiltration. Microscopic invasion by the tumor into surrounding adipose tissue beyond the capsule was found, while there was no evidence of lymph node metastasis. Immunohistochemical analysis revealed tumor cells were positivity for Bcl2, CD5, and p40, while infiltrated lymphocytes were positive for CD3 and negative for CD99 (Fig. 2). EB-encoded RNA in situ hybridization of the tumor was negative. The diagnosis was thymic LELC, classified as p-T1aN0M0 stage I in the TNM classification and stage II according to the Masaoka staging system.

Five months after the operation, follow-up CT findings showed enlargement of lymph nodes in the right supraclavicular fossa, right axillary region, and right mandibular, as well as mediastinal lymph nodes. FDG-PET/CT revealed uptake (SUVmax 6.6) between the chest muscles and axillary lymph node, and needle biopsy results led to a diagnosis of lymph node metastasis from thymic LELC. Chemotherapy was planned for treating metastasis. However, the patient died from another disease at 8 months after the operation.

## Discussion

Shimosato initially reported thymic carcinoma in 1977 as a squamous cell carcinoma of the thymus [4], which has recently been shown to account for 14% of all thymic epithelial tumor cases [3, 4]. According to the 2015 version of the World Health Organization classification [5], there are 12 subtypes of thymic carcinoma, including thymic LELC. This tumor has histologic features similar to lymphoepithelioma, a type of undifferentiated nasopharyngeal carcinoma. According to an ITMIG study, squamous cell carcinoma accounts for 80% of all thymic carcinoma and thymic LELC for 6% [3], while a report from the Japanese Association for Research of the Thymus (JART) noted that thymic LELC accounts for 1.3% of all thymic carcinoma cases in Japan [6]. Kondo et al. reported that thymic LELC accounts for 1.1% of all thymic carcinoma cases in Japan [7]. In our institution, there were only these 2 cases among 43 resected thymic carcinomas (4.7%). Because of these reports, racial differences may influence an incidence of LELC, but there was no report referring to regional differences on thymic LELC.

Thymic LELC has been shown to be related to Epstein-Barr virus (EBV), while EBV has also been linked particularly to LELC of the head and neck, thymus, lung, stomach, and salivary gland [2]. Furthermore, another study reported that over 90% cases of LELC of the head and neck are EBV positive [8]. On the other hand, almost half of thymic LELC cases were found to be positive for EBV [5], lower as compared to LELC of the head and neck. The prognosis of thymic LELC patients is apparently irrespective of the presence or absence of EBV [2]. We performed EB-encoded RNA in situ hybridization of the tumors in both cases, though they were shown to be negative.

It is difficult to diagnose thymic carcinoma including thymic LELC with preoperative CT findings. In case 1, a thymoma was suspected by preoperative CT findings. On the other hand, the tumor in case 2 had accumulation of FDG (SUVmax 6.5) on FDG-PET/CT. Previous reports indicated that a thymic carcinoma showed more increased uptake in FDG-PET than thymoma [9]. But there was no report describing differences in radiological findings of thymic LELC and other subtypes of thymic carcinoma.

We performed a search of other reports of thymic LELC, and clinical manifestations and follow-up data thus obtained are summarized in Table 1 [10–25]. The median survival time (MST) of 24 patients with a prognosis reported was 36 months. A total resection was performed for only 5 of 13 who underwent surgical treatment. Six patients including the present 2 were diagnosed lower than clinical Masaoka stage II, 5 patients were doing well without disease at the latest examination. Of 5 patients who underwent total resection including the 2 in the present report, findings of metastasis in the lung, pleura, and axillary lymph nodes were found in 2. Among all who underwent surgical resection, most cases of recurrence were distant metastasis as compared to local recurrence.

**Table 1:** Previous reports of thymic LELC with a summary of clinical manifestations

	Reference		Age	Sex	Symptom	Clinical Masaoka stage	Treatmemt	Recurrence	Outcome
1	Taylor et al. [10]	1988	43	F	Chest pain, cough	IVb	RT + CTx	–	28 m D
2			28	M	Chest pain	IVb	RT + CTx	–	36 m D
3	Dimery et al. [11]	1988	30	F	Short of breath	IVb	RT + CTx	–	24 m A*
4	Hartmann et al. [12]	1990	38	F	Dyspnoea	IVb	RT + CTx	–	5 m D
5			68	M	Dyspnoea, chest pain, weight loss	–	S + ART	Lung	3 m D
6			46	M	Short of breath	IVb	RT	–	9 m D
7	Kuo et al. [13]	1990	19	M	Chest pain, cough	IVa	S + ACRT	Local recurrence, LN, bone	39 m D
8			41	M	Chest pain	IVb	S + ACRT	Lung, bone	19 m D
9	Fujii et al. [14]	1993	13	F	None	IVb	S + ACRT	Lung, dissemination	22 m D
10	Niehues et al. [15]	1996	14	M	Weight loss	–	S + ACRT	Local recurrence, LN	12y A*
11	Hsu et al. [16]	1998	65	F	Chest pain	–	S + RT	None	6 m A
12	Takahashi et al. [17]	2000	66	F	None	III	S	Lung, dissemination	35 m D
13	Nicolato et al. [18]	2001	55	M	Chest pain, cough	IV	CTx	–	–
14	Yaris et al. [19]	2006	16	F	Cheast pain, dyspnoea	IVb	RT + CTx	–	15 m D
15	Tacyildiz et al. [20]	2007	10	M	Chest pain	III	CTx + S + ACRT	None	12 m A
16	Kilis-Pstrusinska et al. [21]	2008	16	M	Cough	–	CTx	–	11 m D
17	Sekihara et al. [22]	2014	14	M	Chest pain	IVb	CTx	–	10 m D
18	Shima et al. [23]	2016	22	M	Chest pain, dyspnoea	IVa	RT + CTx	–	–
19	Suster et al. [24]	2018	55	M	None	II	S	None	16y A
20			57	F	Fatigue	II	S	None	16y A
21			55	M	None	I	RT	–	8y A*
22			60	M	None	II	No treatment	–	7y A
23			20	M	Cough, short of breath	IV	RT + CTx	–	12 m A*
24			67	F	Chest pain, cough	III	No treatment	–	1 m D
25	Pan et al. [25]	2019	7	M	Short of breath	IVb	S + ACRT	–	–
26	Our cases		65	F	Chest pain	II	S	None	10 m A
27			81	F	None	II	S	LN	7 m D

*A* alive, A* alive with reccurence, *ACRT* adjuvant CRT, *ART* adjuvant RT, *CRT* chemotherapy and radiotherapy, *CTx* chemotherapy, *D* dead, *F* female, *LN* lymph node, *m* months, *M* male, *RT* radiotherapy, *S* surgery, *y* years

Treatment performed after diagnosis of recurrence of thymic LELC is generally radiation therapy and/or chemotherapy, though there is currently no standard protocol established. Chemotherapy for thymic LELC based on treatment for similar types of thymomas or nasopharyngeal carcinomas has been reported [23]. An ADOC (doxorubicin-cisplatin-vincristine-cyclophosphamide) regimen with cisplatin-anthracycline is recommended based on standard thymoma treatment strategies. A phase II study of paclitaxel and carboplatin combination chemotherapy showed that to be effective for nasopharyngeal carcinoma, with an overall response rate of 59%. In our search of other reports, we noticed that thymic LELC cases are often given anthracycline- or platinum-based regimens [10, 11, 19–23]. On the other hand, effects of radiation therapy have not been reported. Previous reports indicate that thymic LELC has a poor prognosis compared to squamous cell carcinoma. But according to the ITMIG database, the survival and recurrence rates are similar to those of other types of thymic carcinoma [5] and surgical treatment is expected to improve prognosis regardless of the type of thymic carcinoma.

Each of the present cases received an early diagnosis and found to be stage II in the Masaoka staging system; thus, surgical resection was performed for both. At the most recent examinations, 1 patient was doing well without disease after undergoing a total resection, whereas the other had early recurrence of distant lymph node metastasis at 5 months after a total resection and died soon thereafter from a different disease. We consider that a successful total resection can positively affect prognosis, though the rate of recurrence rate is high even if complete resection is achieved; thus, long-term follow-up examinations must be performed.

## Conclusion

We treated 2 patients for thymic LELC with surgical resection. Currently, there is no standard treatment strategy for this tumor; thus, additional case reports as well as analysis are needed.

## Data Availability

All data generated or analyzed during this study are included in this published article.

## Abbreviations

ADOC: Doxorubicin-cisplatin-vincristine-cyclophosphamide
CT: Computerized tomography
EBV: Epstein-Barr virus
FDG-PET/CT: 18F-fluorodexyglycose positron emission tomography/computerized tomography
ITMIG: International Thymic Malignancy Interest Group
JART: The Japanese Association for Research of the Thymus
LELC: Thymic lymphoepithelioma-like carcinoma
MST: Median survival time
